# A pyridinium derivative from Red Sea soft corals inhibited voltage-activated potassium conductances and increased excitability of rat cultured sensory neurones

**DOI:** 10.1186/1471-2210-6-10

**Published:** 2006-07-06

**Authors:** Tarek A Temraz, Wael E Houssen, Marcel Jaspars, David R Woolley, Kerrie N Wease, Steven N Davies, Roderick H Scott

**Affiliations:** 1 Marine Science Department, Suez Canal University, Ismailia, Egypt; 2 Marine Natural Products Laboratory, Department of Chemistry, University of Aberdeen, Old Aberdeen, AB24 3UE, Scotland, UK; 3 College of Medical Sciences, Institute of Medical Science, The University of Aberdeen, Foresterhill, Aberdeen AB25 2ZD, Scotland, U.K

## Abstract

**Background:**

Whole cell patch clamp recording and intracellular Ca^2+ ^imaging were carried out on rat cultured dorsal root ganglion (DRG) neurones to characterize the actions of crude extracts and purified samples from Red Sea soft corals. The aim of the project was to identify compounds that would alter the excitability of DRG neurones.

**Results:**

Crude extracts of *Sarcophyton glaucum *and *Lobophyton crassum *attenuated spike frequency adaptation causing DRG neurones to switch from firing single action potentials to multiple firing. The increase in excitability was associated with enhanced KCl-evoked Ca^2+ ^influx. The mechanism of action of the natural products in the samples from the soft corals involved inhibition of voltage-activated K^+ ^currents. An active component of the crude marine samples was identified as 3-carboxy-1-methyl pyridinium (trigonelline). Application of synthetic 3-carboxy-1-methyl pyridinium at high concentration (0.1 mM) also induced multiple firing and reduced voltage-activated K^+ ^current. The changes in excitability of DRG neurones induced by 3-carboxy-1-methyl pyridinium suggest that this compound contributes to the bioactivity produced by the crude extracts from two soft corals.

**Conclusion:**

*Sarcophyton glaucum *and *Lobophyton crassum *contain natural products including 3-carboxy-1-methyl pyridinium that increase the excitability of DRG neurones. We speculate that in addition to developmental control and osmoregulation these compounds may contribute to chemical defenses.

## Background

The potential benefits of marine pharmacology remain to be fully realised. Diverse and novel natural products isolated from bacteria, algae and benthic invertebrates including soft corals, sponges and anemones from distinct marine environments have been chemically identified and some of their biological activities characterised [1]. Marine organisms may contain many potential novel drugs because of the unique environmental conditions (high ionic strengths, low light level, cold or warm temperatures, and pressure) found in their habitats. These conditions have led to the biosynthesis of unique compounds [2]. However, such studies are associated with clear difficulties that include the taxonomy of organisms, identification of the origins of bioactive materials, reproducibility of material collection and complex chemistry [3].

The Red Sea has two major distinctive features. It has one highest levels of marine bio-diversity and it has great seasonal fluctuations of air and water temperatures. Conducting research on Red Sea organisms offers unique advantages, in view of the diversity and high endemism of its biota. For example, of the 180 known species of the soft corals, about 40 % are unique to the Red Sea [4].

Several groups of marine organisms including soft-bodied sessile invertebrates, such as tunicates, soft corals, and certain sponges appear defenceless yet they have few predators and are not substrates for fouling micro-organisms. These organisms are rich in nutritionally important substances and use an arsenal of chemical defences and chemical repellents to protect themselves and when competing for space. The incidence of predation is low because of the production of toxic compounds and the possession of some form of calcareous sclerites [5].

In this study we have used the electrophysiological properties of cultured sensory neurones from rat dorsal root ganglia (DRG) as an assay system to identify and characterize the biological activities on ion channel currents of crude extracts and an active purified common compound present in two soft coral samples. The activities of some soft coral toxins are consistent with pore-formation as a mechanism of action and the natural products responsible may be similar to polymeric alkylpyridinium salts from marine sponges [6-9].

Previously, a number of interesting biologically active compounds from soft corals have been studied and these genera are sources of hundreds of different compounds [2]. A few examples include: 1. Sarcophytolide (lactone cembrane diterpene) from *Sarcophyton glaucum*, which, is antimicrobial and suppresses glutamate-evoked Ca^2+ ^responses and neurone death [10]. 2. Brominated oxylipins from *Dendronephthya spp *and *Tubipora musica*, which are toxic to shrimps, sea urchin eggs and crown gall tumors [11]. 3. Singardin a heptacyclic norcembranoid dimer, which shows cytotoxicity against murine leukemia, human melanoma cells, human lung and colon carcinomas [12]. 4. Lophotoxins from a variety of soft corals that are nicotinic acetylchoine receptors antagonists [13,14]. 5. Palytoxin, from the genus *Palythoa*, which is highly poisonous and increases membrane permeability to cations and potently inhibits Na^+^/K^+ ^ATPase [15]. 6. A C-29 steroid from *Lobophytum crassum *the biological activity of which is yet to be reported [16]. Ethyl acetate extraction of a single species of soft coral (*Lobophytum catalai *Tixier-Durivault) has yielded a number of distinct novel compounds (two cembranoids, nephthenol, furanosesquiterpene, four polyhydroxysterols and mixtures of sesquiterpenes and monohydroxysterols) [17]. The many diverse compounds are produced, some within nematocyst venoms that have hemolytic, dermonecrotic and vasopermeabilising factors [18], others are confined to protective mucus [19], eggs and larvae (pukalide and 11 β-acetoxypukalide), or are released into the water column during mass spawning [20].

Although we have failed to find a pore forming compound, our research has identified a natural product that can dramatically increase the excitability of cultured sensory neurones and therefore may act as a pronociceptive agent against predators.

## Results and discussion

### Chemical characterization of the soft coral samples and identification of the active compound

**Figure 1:**
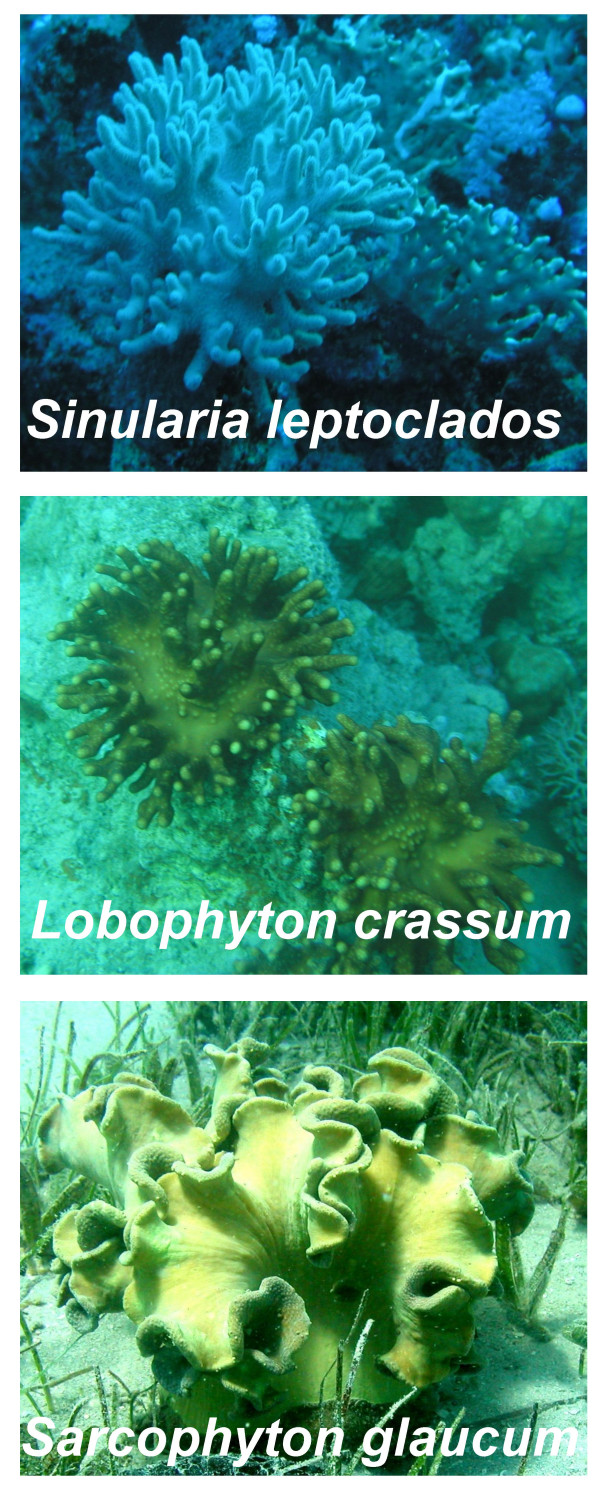
Underwater photographs of the species soft corals used in this study.

Figure 1 shows pictures of the three species of Red Sea soft corals (*Sarcophyton glaucum, Lobophyton crassum & Sinularia leptoclados*), which were the sources of the natural product materials used in this study.

**Figure 2:**
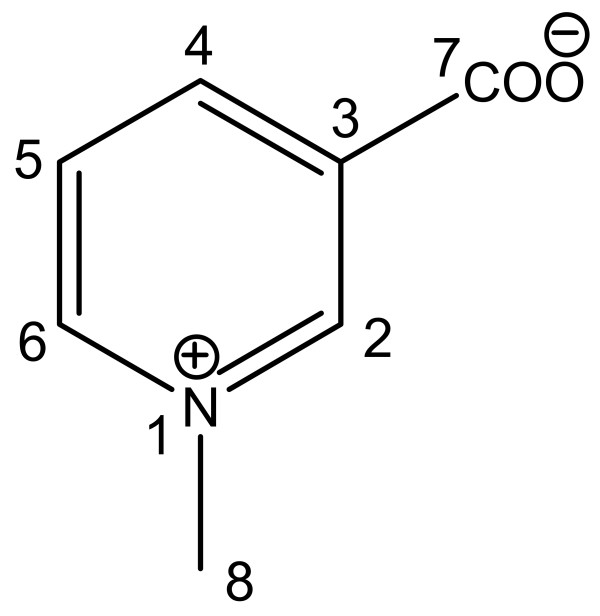
Structure of 3-carboxy-1-methyl pyridinium (CMP; trigonelline).

The similarity of the ^1^H NMR spectra of crude extracts, especially in the region 7–10 ppm, from *Sarcophyton glaucum *and *Lobophyton crassum *provided the starting point for comparison of bioactivity. The specimens of *Sarcophyton glaucum *and *Lobophyton crassum *were not heavily armoured but were free from fouling organisms indicating that they had efficient chemical defenses. In contrast, *Sinularia leptoclados *is heavily armoured but does not contain material with a similar ^1^H NMR spectrum as seen for the other two species. Table 1 shows the NMR data for 3-carboxy-1-methyl pyridinium (Fig. 2) obtained in CD_3_OD. The low-resolution electron impact mass spectrometry (LREIMS) of 3-carboxy-1-methyl pyridinium showed a pseudomolecular ion [M + H]^+ ^at *m/z *138. The high-resolution electron impact mass spectrometry (HREIMS) provided the exact value of *m/z *138.0549 (Δ 0.1 mmu) for [M + H]^+^, which corresponded well with the formula C_7_H_7_NO_2_. The formula suggested five degrees of unsaturation. The ^1^H NMR spectrum of 3-carboxy-1-methyl pyridinium exhibited signals at δ_H _9.33 (1H, s), 8.96 (1H, s), 8.95 (1H, s), and 8.11 (1H, bt), whose chemical shifts were reminiscent of those of some pyridine mesomeric betaine alkaloids e.g. pyridinebetaine A [21], and homarine [22]. These values were assigned to H-2, H-6, H-4 and H-5 protons respectively and their connectivities to their respective carbons (δ_C _147.0, CH; 145.2, CH; 147.0, CH and 127.7, CH) were determined from a heteronuclear single quantum correlated spectroscopy (HSQC) experiment. The ^1^H NMR spectrum also exhibited a proton singlet at δ_H _4.43 (3H), which showed heteronuclear multiple bond correlated spectroscopy (HMBC) correlations with C-2 (δ_C _147.0, CH) and C-6 (δ_C _145.2, CH). This evidence indicated the presence of a methyl group on N-1.

**Table 1:** 1D and 2D NMR spectral data for 3-carboxy-1-methyl pyridinium obtained in CD_3_OD (δ in ppm).

No.	δ_C_		δ_H _(#H, m, *J*/Hz)	^1^H-^1^H COSY	HMBC (δ_C _to δ_H_)
2	147.0	CH	9.33 (1H, s)	H-6, H-8	H-4, H-6, H-8
3	162.0	qC			
4	147.0	CH	8.95 (1H, s)	H-5	H-2, H-5, H6
5	127.7	CH	8.11 (1H, bt)	H-4, H-6	H-4, H-6
6	145.2	CH	8.96 (1H, s)	H-2, H-5, H-8	H-2, H-4, H-8
7	166.0	qC			
8	47.6	CH_3_	4.43 (3H, s)	H-2, H-6	H-6

Further database searches led to the identification of the structure as the long-known alkaloid, 3-carboxy-1-methyl pyridinium (trigonelline). Confirmation of the structure was carried out by comparison of our NMR data with spectra from an authentic sample of 3-carboxy-1-methyl pyridinium (trigonelline hydrochloride) as well as with data from a previous study [23].

### Actions of crude samples from soft corals on cultured DRG neurone action potential properties

All the experiments conducted in this study were carried out in the continual presence of extracellular recording solution that contained 0.1% DMSO. This concentration of DMSO was required to keep the crude extracts from Red Sea soft corals in solution. Under these recording conditions the mean resting membrane potential and input resistance (derived from electrotonic potentials produced by -100 pA step commands) were -61 ± 2 mV (n = 35) and 338 ± 45 MΩ (n = 16) respectively. These values are similar to the mean values (-60 mV and 356 MΩ; n = 12), we have previously reported from studies carried out on cultured DRG neurones in the absence of DMSO [6]. DRG neurones are a heterogeneous population of neurones, which show a variety of distinct structural, biochemical and electrophysiological characteristics. To standardize the electrophysiological recordings made from DRG neurones the cells were held at a potential of -70 mV and depolarized with 100–800 ms current step commands to activate action potentials. Most of our cultured DRG neurones (~90%; n = 31 of 34) show spike frequency adaptation (accommodation) and fire only a single action potential in response to a prolonged supra-maximal depolarizing current command. In the absence of DMSO we have reported multiple action potential firing in less than 20% of DRG neurones [24].

The recording method, animal species and culture conditions may influence multiple firing levels in sensory neurones and the incidence is higher in some other studies. For example previous work on whole nodose ganglia and ganglia slices showed that 38.5% of C-fibre neurones and 66.7% of A-fibre neurones show multiple firing properties [25].

**Figure 3:**
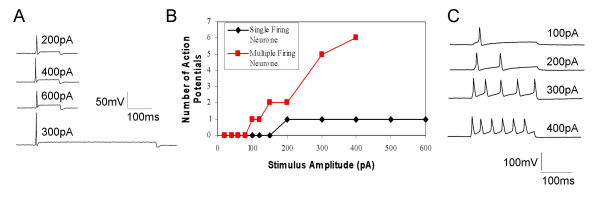
Comparison of electrophysiological activity between a single firing DRG neurone and a multiple firing DRG neurone. A) Traces showing the single action potential firing of a DRG, stimulated with 200, 400 and 600 pA depolarizing step commands and a prolonged 500 ms stimulus. B) Line graph illustrating the effect of increasing current commands on both single (blue line) and multiple action potential firing (red line) DRG neurones. C) Traces showing the effects of increasing stimulus amplitude (100–400 pA) on a multiple action potential firing DRG neurone. An increase in stimulus amplitude leads to an increase in the number of action potentials. In all cases the DRG neurones were held at -70 mV before stimulation.

Increasing the duration or amplitude of the stimulus does not increase the firing frequency in neurones that show spike frequency adaptation (Fig. 3A &3B). However, some cultured DRG neurones do not show spike frequency adaptation and fire increasing numbers of action potentials at higher frequencies as the amplitudes of current step commands are increased (Fig. 3B &3C).

**Figure 4:**
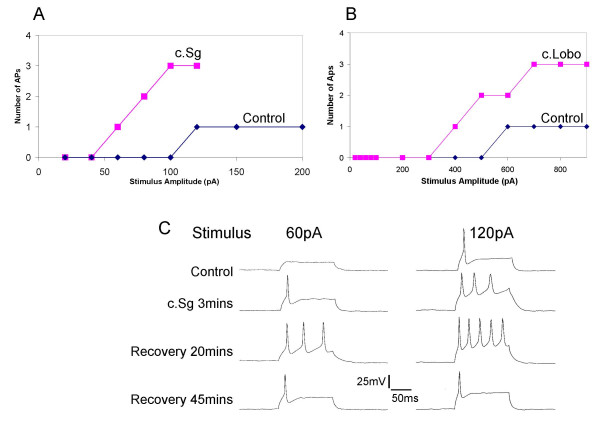
Application of c.Sg or c.Lobo evoked a switch from single action potential firing to multiple firing in DRG neurones. A) Line graph showing that prior to application of c.Sg (100 μg/mL) only one action potential is evoked over a range of supramaximal stimuli (blue line). Application of c.Sg caused a reduction in threshold stimulus for action potential firing as well as a switch from single to multiple firing (red line). B) Similarly, c.Lobo (100 μg/mL) reduced the threshold for firing and converted a single firing DRG neurone (blue line) to a multiple firing neurone (red line). C) Current clamp traces illustrate the multiple firing behaviour of neurones that have been exposed to c.Sg. Also seen is the ability of c.Sg to reduce the threshold for firing. Furthermore, recovery is shown 45 minutes after the pressure ejecting c.Sg. In all cases the DRG neurones were held at -70 mV before stimulation.

Application of a crude sample from *Sarcophyton glaucum *(c.Sg; 100 μg/mL) for 3–5 minutes induced a dramatic increase in action potential firing. In six neurones that showed spike frequency adaptation, c.Sg increased the number of action potentials evoked during 100 ms supra-maximal current commands from 1 action potential to a mean of 4 ± 1 (n = 6; *P *< 0.05). The number of action potentials was dependent on the amplitude and duration of the stimulus (Fig. 4A). Similarly, application of a crude sample from *Lobophyton crassum *(c.Lobo; 100 μg/mL) for 3–5 minutes also induced increases in action potential firing (Fig. 4B). In five neurones that showed spike frequency adaptation under control conditions, c.Lobo increased the number of action potentials evoked during 100 ms supra-maximal current commands from 1 action potential to a mean of 3 ± 1 (n = 5; *P *< 0.03). Furthermore, both c.Sg and c.Lobo samples reduced the amplitudes of the current stimuli required for the thresholds of action potential firing to be reached (Figure 4A &4B).

These responses developed gradually and in some cases continued to develop over 20 minutes after perfusion of the crude sample was stopped. However, recovery from these effects was seen after 45 minutes and reapplication of either of the crude soft coral extracts could produce repeatable responses. Figure 4C illustrates some example records of action potential firing patterns evoked by +60 pA and +120 pA step commands, which show the features of the crude samples actions. These actions included an increased sensitivity to depolarizing current and a reversible lose of spike frequency adaptation. These effects were produced without any significant change in the resting membrane potential.

**Figure 5:**
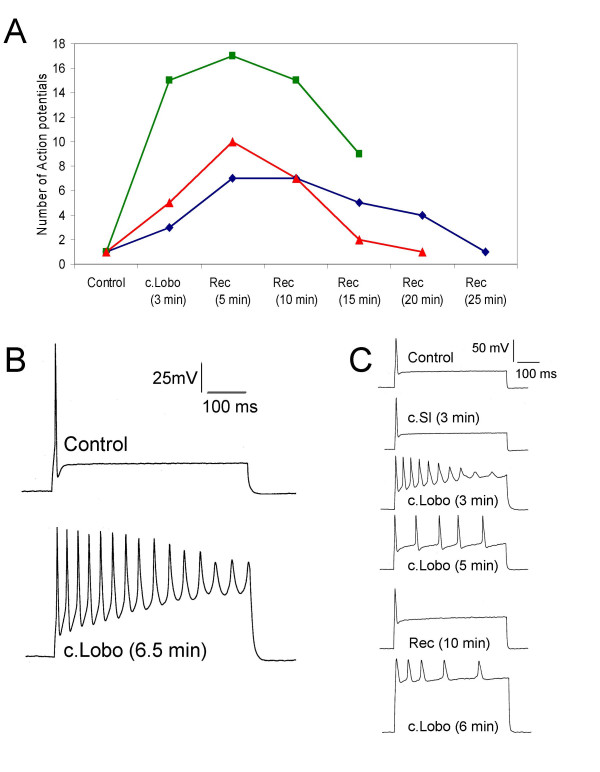
The effect of c.Lobo and c.Sl on cultured DRG neurones. A) Time course showing the action of c.Lobo (100 μg/mL) in three separate DRG neurones stimulated with 500 ms current step commands. Extracellular application of c.Lobo leads to an increase in the number of action potentials evoked relative to the control. After removal of the drug pipette, an increase in action potential number is followed by a steady decrease to basal levels. B) Voltage traces illustrating the effects that c.Lobo has on both action potential number and action potential shape. C) Voltage traces showing the inability of c.Sl to evoke a switch to multiple firing, followed by application of c.Lobo, which initiates multiple firing. For all records the DRG neurone was stimulated with depolarizing 600 pA step commands. Recovery is seen after removal of c.Lobo and upon further application multiple firing is once again evoked. In all cases the DRG neurones were held at -70 mV before stimulation. with 500 ms.

Increasing the duration of the stimulus to 500 ms revealed further characteristics of the excitatory actions of the crude soft coral samples. Figure 5A shows the diversity of the maximum levels of excitation achieved following three minutes application of 100 μg/mL c.Lobo that continued to develop during 5 minutes recovery. In these cases 400–600 pA depolarizing currents activated between 7 and 15 action potentials in 500 ms. However, the features of the multiple firing induced by c.Lobo were characterized by a gradual decline in action potential amplitude, a progressive failure in membrane potential repolarization and a gradual upward drift in the depolarizing electrotonic potential (Fig. 5B). In some cases, the drift in the depolarizing electrotonic potential was not dependent on action potentials but could also be seen to produce unusual irregular firing patterns where action potentials were observed only at the beginning and end of the stimulus. The striking effects on action potential firing of c.Sg and c.Lobo, which had similar ^1^H NMR spectra (region 7–10 ppm), were not seen when 100 μg/mL of crude sample from *Sinularia leptoclados *(c.Sl) was applied to DRG neurones. The same neurones that were unaffected by the sample from *Sinularia leptoclados *were sensitive to repeated application of c.Lobo (Fig. 5C). These data suggest that chemical entities common to both c.Sg and c.Lobo and possibly detected by NMR may be responsible for their actions on DRG action potentials.

**Figure 6:**
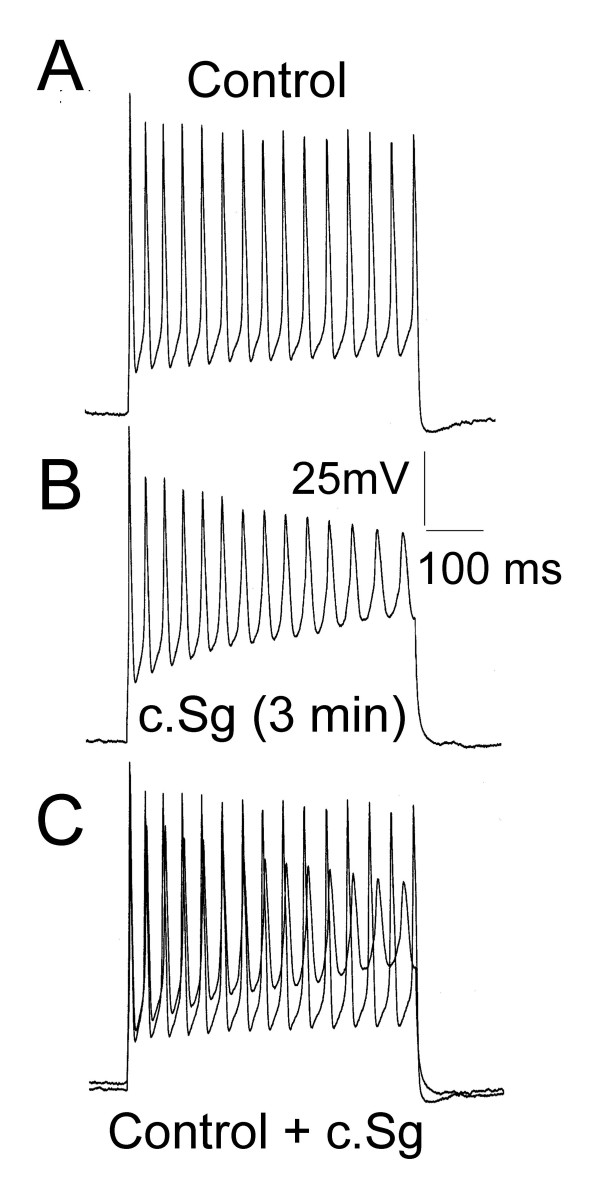
Some DRG neurones under control conditions do not show spike frequency adaptation but fire multiple action potentials during depolarization by 500 ms current commands. This was a different firing pattern to that seen when c.Sg induced multiple fire. A) Voltage trace of control multiple action potential firing neurone. B) Voltage trace of c.Sg-induced multiple firing in the same neurone. C) Over-plot of voltage traces from A and B. In all cases the DRG neurones were held at -70 mV before stimulation.

The multiple action potential firing patterns induced by c.Sg and c.Lobo were distinct from the control multiple firing observed in a small proportion of DRG neurones. Figure 6 shows example records of the actions of c.Sg (100 μg/mL) on a DRG neurone, which under control conditions showed multiple firing. Three minutes application of c.Sg did not attenuate action potential firing but resulted in a gradual decline in action potential amplitude, an increase in action potential duration and a progressive failure in complete action potential re-polarization.

**Figure 7:**
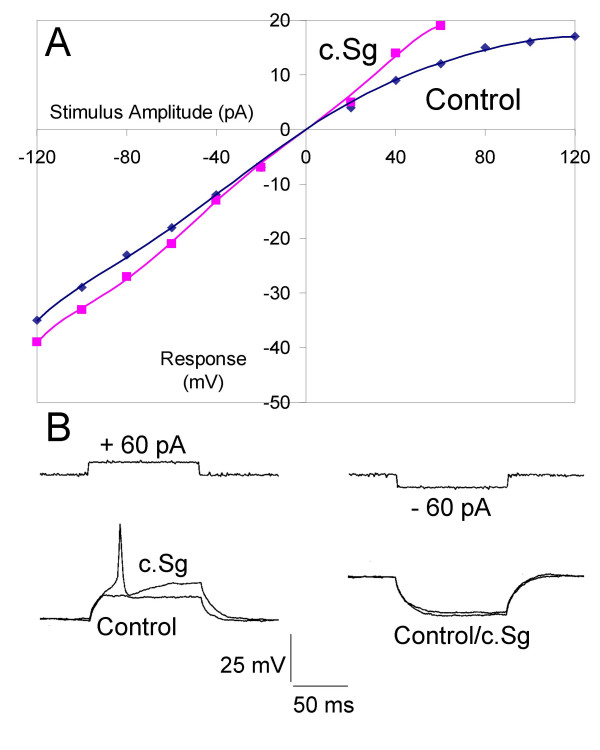
Rectification in DRG neurones is attenuated by c.Sg in DRG neurones. A) Line graph showing voltage-current relationships before (Control blue line) and after the application of c.Sg (100 μg/mL; red line) in the same neurone. B) Traces of current commands +60 pA and -60 pA and voltage responses in cultured DRG neurone before and after the application of c.Sg. In all cases the DRG neurones were held at -70 mV before stimulation.

The electrotonic responses to depolarizing current commands indicated that c.Sg and c.Lobo may influence rectification in DRG neurones. This was investigated by generating current-voltage relationships in the presence and absence of 100 μg/mL c.Sg (Fig. 7A) or 100 μg/mL c.Lobo. Three to five minutes application of c.Sg (n = 4) or c.Lobo (n = 4) had no significant effect on the mean electrotonic potential produced by -60 pA (combined control -15 ± 3 mV; crude sample -17 ± 3 mV (n = 8; NS)). In contrast the mean depolarizing electrotonic potential produced by +60 pA step commands increased, an indication of a reduction in the rectification (Fig. 7B). Under control conditions the mean electrotonic potential was 8 ± 1 mV and in the presence of c.Sg or c.Lobo the combined value increased to 11 ± 2 mV (n = 8; *P *< 0.005).

The changes in action potential firing properties induced by soft coral extracts could have resulted from modulation of voltage-activated Na^+ ^channels. To investigate this we studied the properties of single action potentials evoked by 5 ms depolarizing currents. Action potential firing threshold and amplitude were not significantly altered by the soft coral extracts (100 μg/ml). Under control conditions and in the presence of soft coral extracts the threshold for firing (-34 ± 1 mV & -35 ± 1 mV) and peak action potential amplitude (31 ± 2 mV & 34 ± 2 mV; n = 12) were unchanged. However, the mean action potential duration at 50% of peak amplitude was increased from 2 ± 0.1 ms to 2.8 ± 0.1 ms (n = 12; P < 0.005) by the soft coral extracts.

Given the effects of two crude extracts on action potential duration and firing we decided to investigate whether the increases in excitatory properties of DRG neurones were accompanied by increases in intracellular Ca^2+ ^concentration evoked by depolarization.

### Actions of crude extracts from soft corals on intracellular calcium in DRG neurones

**Figure 8:**
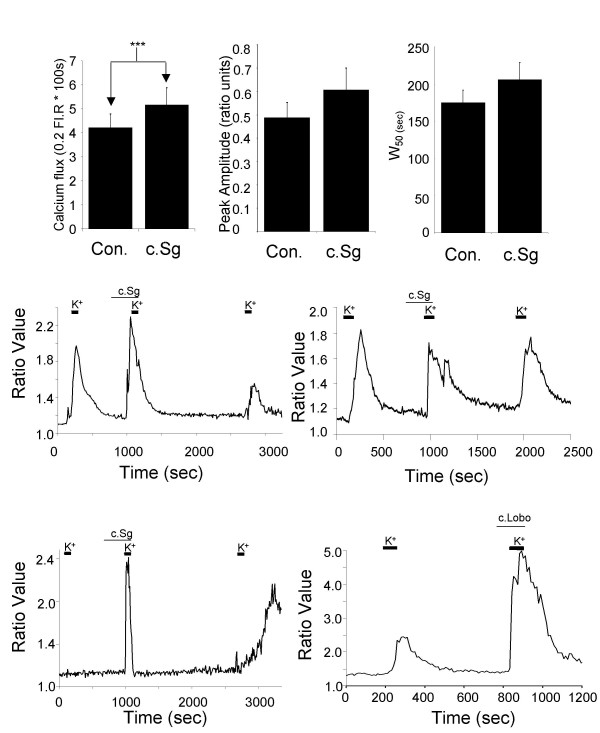
Application of c.Sg increases K^+^-evoked calcium flux into cultured DRG neurones. A) Bar chart showing a significant enhancement of the K^+^-evoked Ca^2+ ^flux induced by c.Sg compared to the control (Con.). B) Bar chart showing mean peak Ca^2+ ^transient amplitudes evoked by K^+ ^under control conditions (Con.) and in the presence of c.Sg. C) Bar chart showing mean values for the width 50% of the Ca^2+ ^transient evoked under control conditions (Con.) and in the presence of c.Sg. For A, B and C mean values ± SEM are shown from 17 neurones (***P < 0.005). D) Example trace of Ca^2+ ^transients showing the increase in amplitude in a single neurone exposed to c.Sg. E) Example trace showing enhancement in Ca^2+ ^transient duration in a single neurone exposed to c.Sg. F) Trace demonstrating that application of c.Sg can cause K^+^-evoked Ca^2+ ^flux in a neurone that previously showed no K^+^-evoked Ca^2+ ^flux. G) Example trace showing that c.Lobo has a similar effect to c.Sg. In all traces (D to G) the period of stimulation with KCl (30 mM) is shown by thick bars and the period of application of crude samples from soft corals is shown with thin bars.

The crude soft coral extracts also modulated increases in intracellular Ca^2+ ^evoked by KCl (30 mM) stimulation. In particular, both c.Sg and c.Lobo increased KCl-evoked Ca^2+ ^transients and in a subpopulation of DRG neurones the extracts reduced the threshold for activating a Ca^2+ ^transient (Fig. 8). Detailed analysis of the Ca^2+ ^transients evoked by KCl showed that total Ca^2+ ^flux was increased significantly (n = 10) by 100 μg/mL c.Sg (Fig. 8A). Despite this, the nature of the enhancement in individual neurones varied such that there was no significant increase in the mean peak amplitude (Fig. 8B) or width of the response at 50% of peak amplitude (W_50_; Fig. 8C). In some cases the peak Ca^2+ ^transient amplitudes were clearly increased by c.Sg (Fig. 8D) but in other neurones the amplitudes were not increased but the durations of the responses were increased and in some instances appeared to show multiple peaks (Fig. 8E). The actions of c.Sg on Ca^2+ ^transients appeared to be reversible (Fig. 8D &8E). However, some caution is necessary in the interpretation of the results as the decline in the third response to KCl may result from Ca^2+^-induced inactivation of voltage-activated Ca^2+ ^channels brought about by the increased intracellular Ca^2+ ^loads. Figure 8F shows an example record from a DRG neurone that did not respond to 30 mM KCl stimulation initially but subsequently responded to KCl when applied in the presence of c.Sg. This phenomena occurred in 14 out of 24 DRG neurones and interestingly these neurones responded to a third KCl pulse applied alone after priming with c.Sg. This data is of particular concern as it suggests that stimulation with 30 mM KCl may not be sufficient to activate Ca^2+ ^transients in all cultured DRG neurones. Previously, we found that 30 mM KCl depolarizes DRG neurones by 25 to 30 mV. Stimulation with 30 mM KCl allows consistent Ca^2+ ^transients to be activated though it may bias analysis towards DRG neurones with low thresholds for excitation.

The cultured DRG neurones investigated in this study had a mean cell body area of 256 μm^2 ^(n = 10; range 124 to 410 μm^2^). Responses to c.Sg. were obtained from neurones with different sizes of cell body and different sensitivities to the natural product preparation did not appear to relate to size of cell body.

Similar results were obtained with c.Lobo (100 μg/mL; n = 4). Figure 8G shows a striking example of enhancement of a KCl-evoked Ca^2+ ^transient in the presence of c.Lobo, but a more complete analysis of this sample was not possible because of the limited amount of material available.

### Actions of crude extracts from soft corals on voltage-activated potassium currents in DRG neurones

**Figure 9:**
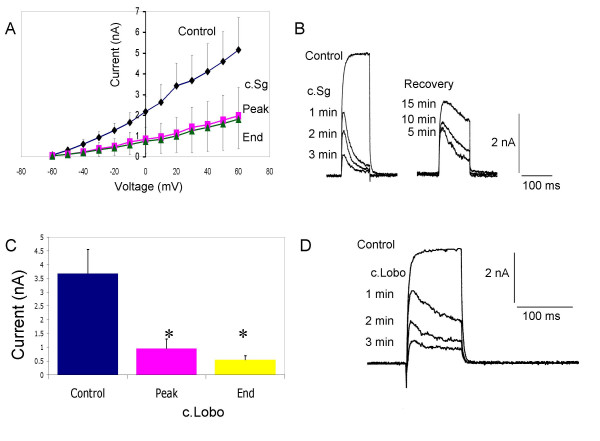
Whole cell voltage-activated K^+ ^currents in cultured DRG neurones are significantly reduced by c.Sg or c.Lobo (100 μg/mL). A) Line graph showing mean (± sem) current-voltage relationships under control conditions (blue line) and after the application of c.Sg (red and green lines). The current amplitudes measured at the peak (red) and at the end of the voltage step command (green) are illustrated. B) Net K^+ ^current traces showing the onset of the inhibitory effects of c.Sg and the time course of partial recovery. The neurone was voltage clamped at -70 mV and currents were evoked by voltage step commands to +60 mV. C) Bar chart illustrating mean current amplitudes (n = 6; *P < 0.02) at +60 mV, under control conditions (blue bar), the peak current in the presence of c.Lobo (red bar) and the current recorded at the end of a 100 ms voltage step command in the presence of c.Lobo (yellow bar). D) Net K^+ ^current traces showing the development of current inhibition by c.Lobo. The neurone was voltage clamped at -70 mV and currents were evoked by voltage step commands to +60 mV.

The effects of the crude soft coral extracts suggested their action, at least part, involved the inhibition of voltage-activated K^+ ^conductances. Specifically, the soft coral samples from *Sarcophyton glaucum *and *Lobophyton crassum *prolonged action potentials, attenuated spike frequency adaptation, reduced rectification and enhanced KCl-evoked Ca^2+ ^transients, all of which could be associated with inhibition of K^+ ^currents. DRG neurones express diverse K^+ ^channels and in small DRG neurones five types have been identified (A, DR_F_, DR_1_, DR_2 _& DR_3_). All these channels play roles in the control of action potential repolarisation and DR_1–3 _influence action potential firing threshold and after-hyperpolarisation and thus can influence firing patterns [26]. To investigate the actions of the soft coral extracts further, from a holding potential of -70 mV, outward currents were activated by 100 ms voltage step commands to potentials positive to -50 mV. Three to five minutes application of 100 μg/mL c.Sg significantly inhibited the voltage-activated K^+ ^current at all voltages between -40 mV and +60 mV (Fig. 9A &9B).

Control currents were activated fully within 15 ms and were sustained during 100 ms depolarizations, but in the presence of c.Sg the currents shape was changed. After 1 minute application of c.Sg the outward currents were reduced and became transient in nature. Partial recovery of the outward current was observed 15 minutes after removal of the perfusion pipette containing c.Sg. Additionally, partial recovery was also associated with a progressive slowing in the current decay during the voltage step command (Fig. 9B).

The actions of c.Lobo on voltage-activated K^+ ^currents followed a similar pattern to that seen with c.Sg. Application of 100 μg/mL c.Lobo significantly inhibited the voltage-activated outward current and resulted in a decline in the current during the 100 ms voltage step command (Fig. 9C &9D). c.Lobo significantly reduced the outward current at all potentials between -20 mV and +90 mV. At a clamp potential of +60 mV, under control conditions the mean peak outward current was 3.8 ± 0.8 nA and the mean current was significantly reduced to 0.7 ± 0.12 nA (n = 6; *P *< 0.005) by c.Lobo.

The crude extracts from soft corals did not significantly alter linear leak currents activated between -80 and -140 mV. At -120 mV the mean leak currents were -0.026 ± 0.01 nA and -0.032 ± 0.01 nA (n = 9, NS) under control conditions and in the presence of soft coral extract respectively.

The changes in shape of the voltage-activated K^+ ^currents that were particularly apparent during the development of inhibition produced by both soft coral samples may be indicative of an open channel block mechanism of the active natural product common to both crude extracts.

**Figure 10:**
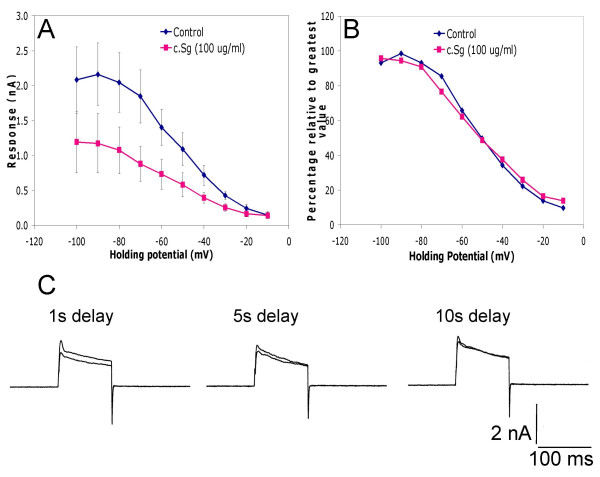
Extract from *Sarcophyton glaucum *(100 μg/mL) did not cause a change in steady state inactivation but does show some activity dependence. A) Line graph showing mean control steady state currents (± s.e.m) activated from holding potentials between -100 and -10 mV by voltage step commands to +30 mV (blue) and the steady state inactivation relationship after application of c.Sg (n = 6). B) Line graph showing the normalised mean steady state inactivation plots under control conditions (blue) and after the application of c.Sg (n = 6). For each plot the current values were normalized with respect to the maximum outward current. C) The effect of a 1 s, 5 s and 10 s delays between concurrent voltage step commands on the net K^+ ^outward current (at + 30 mV) during application of c. Sg (100 μg/mL).

To investigate the mechanism of inhibition we looked for any effect of the soft coral extracts on steady state inactivation and for evidence of use-dependence during the onset of inhibition by crude soft coral extracts (Figure 10). Steady-state inactivation plots allowed the proportion of voltage-gated channels available to open at any given voltage to be determined. Drug interactions with channels or differential effects of a drug to alter the population of active channels can shift steady-state inactivation plots. Under voltage-clamp conditions neurones were held between -100 and -10 mV. From these holding potentials, neurones were stimulated with voltage step commands to +30 mV in the absence (control) and presence of 100 μg/mL c. Sg (n = 6). As can be seen from the raw current data (Fig. 10A) and the normalized plot (Fig. 10B) c. Sg had no significant effects on K^+ ^current steady-state inactivation. The mean voltages at which 50 % of the channels were available to open (V_0.5(inact)_) were -50 ± 2 mV in the control and -53 ± 3 mV (n = 6; NS) in the presence of c. Sg.

Preliminary results indicate that the delay between command steps has a critical effect on the size of the second response when two pulses are given in succession in the presence of c. Sg. A series of experiments were conducted whereby the delays between pulses were varied (1, 5 and 10 seconds). A 10 second delays between step commands resulted in recovery of the K^+ ^current. In contrast a delay of 1 s resulted in further decline in the current (Fig. 10C). These data suggest there is a component of use-dependent inhibition but recovery can occur in 5–10 s. We conclude that the slowly developing main component of K^+ ^current inhibition produced by the soft coral extracts involves another mechanism.

### Actions of purified active compound from *Sarcophyton glaucum*

**Figure 11:**
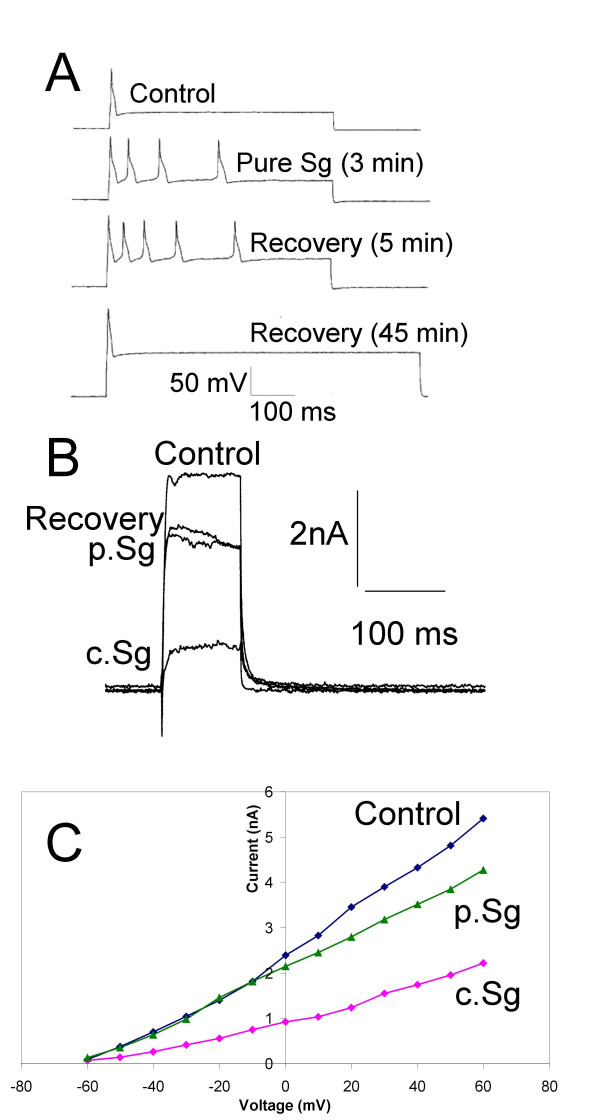
A purified sample from *Sarcophyton glaucum *(p.Sg) increased excitability and attenuated voltage-activated K^+ ^currents in cultured DRG neurones. A) Example of voltage traces showing reversible inhibition of spike frequency adaptation and the development of multiple action potential firing by p.Sg (~10 μM). B) Voltage-clamp current traces showing the inhibitory effects of c.Sg (100 μg/mL) and p.Sg (30 μg/mL; ~200 μM) on DRG neurones held at -70 mV and depolarized to +60 mV by voltage step commands. Partial recovery of the net K^+ ^current was seen after 10 mins. C) Line graph showing the current-voltage relationships under control conditions (blue line), after application of p.Sg (30 μg/mL; ~200 μM; green line) and then c.Sg (100 μg/mL; red line) recorded from a single DRG neurone.

Directed by chemical analysis and biological testing of crude extracts, the common active compound was identified as 3-carboxy-1-methyl pyridinium (trigonelline hydrochloride). This was purified from the crude extracts from *Sarcophyton glaucum *(p.Sg) and tested using electrophysiological protocols described above. DRG neurones were held at -70 mV and bathed in extracellular recording medium containing 0.1% DMSO. Similar to the crude extracts the purified preparation p.Sg (estimated at 30 μg/mL; ~200 μM) attenuated spike frequency adaptation and three minutes application of pure compound (p.Sg) resulted in multiple action potential firing in response to depolarizing current. This response was reversible but recovery was slow and as seen with the crude samples the effect of the natural product deepened after removal of the drug perfusion pipette (Fig. 11A). Similarly, p.Sg also attenuated the mean peak voltage-active K^+ ^currents at +60 mV by 15% (n = 4; *P *< 0.01). However, these responses were modest and further inhibition could be obtained with c.Sg (Fig. 11B &11C). This was in part due to differences in concentrations tested and the small amounts of purified compound available. However, other components within the crude extract may enhance the activity of the major active component from *Sarcophyton glaucum*.

### An investigation of the possible roles of intracellular signalling in the bioactivity of crude extracts from *Sarcophyton glaucum*

**Figure 12:**
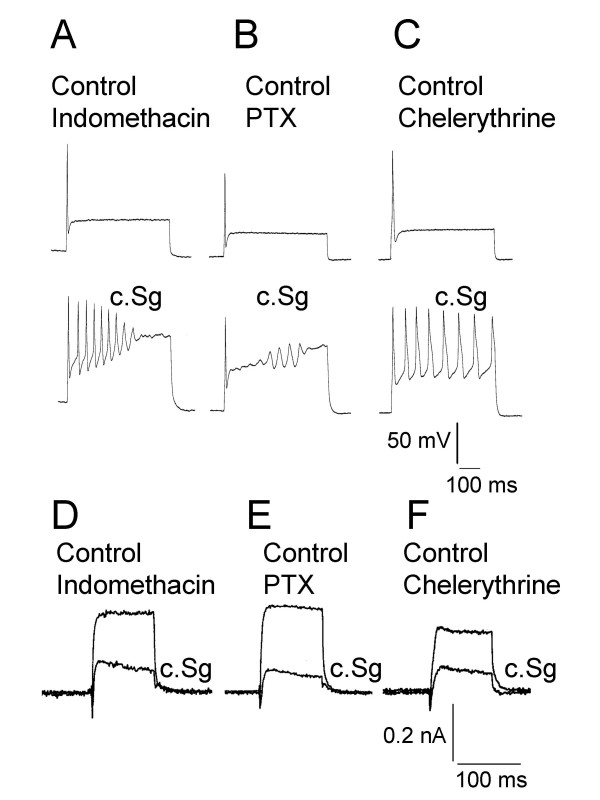
Actions of c.Sg are not inhibited by indomethacin, pertussis toxin pre-treatment or chelerythrine. A, B & C) Voltage records showing action potential firing properties in the presence of the three test treatments (indomethacin, pertussis toxin pre-treatment or chelerythrine) and multiple action potential firing evoked by 100 μg/mL c.Sg. Indomethacin (A) pertussis toxin pretreatment (B) and chelerythrine (C) all failed to prevent the switch to multiple firing after application of c.Sg to DRG neurones that previously fired single action potentials. At rest all neurones were held at -70 mV. D, E & F) Current traces illustrating voltage-activated K^+ ^current activated at +60 mV by 100 ms voltage step commands from a holding potential of -70 mV. In the presence of the three test treatments (indomethacin, pertussis toxin pre-treatment or chelerythrine) c.Sg still induced multiple action potential firing and attenuated the voltage-activated K^+ ^currents.

Slow onset of the responses to the crude extracts from *Sarcophyton glaucum *and *Lobophyton crassum*, the continued development of their effects after it was no longer being applied, and the slow recovery phase all raised the possibility that modulation of neuronal excitability might involve the activation of intracellular signals rather than a direct interaction with K^+ ^channels. To investigate this possibility, three approaches were taken to disrupt certain intracellular signalling which might modulate K^+ ^channels. Firstly, the cyclooxygenase 1 and 2 inhibitor indomethacine (10 μM) was applied to the bath and to the intracellular environment via the patch pipette solution. The activities of cyclooxygenases have been implicated in modulating K^+ ^conductances to increase neuronal excitability [27,28]. Secondly, pertussis toxin sensitive G-proteins were inhibited by incubating the DRG neurones for 18 hours with 500 ng/mL pertussis toxin. Thirdly, the protein kinase C inhibitor chelerythrine (3 μM; [29]) was applied to the intracellular environment via the patch pipette solution. At a concentration of 100 μg/mL, c.Sg persisted in enhancing DRG neurone excitability in the presence of indomethacine, after pertussis toxin pre-treatment and in the presence of chelerythrine. Spike frequency adaptation was attenuated and multiple firing was observed under all three conditions (Fig. 12A,B, &12C). Additionally, as previously observed in the absence of blockers c.Sg inhibited voltage-activated K^+ ^currents in the presence of indomethacin (n = 2; Fig. 12D), after pertussis toxin treatment (n = 3; Fig. 12E) and in the presence of chelerythrine (n = 3; Fig. 12F). Although not an exhaustive list of potential target sites, our data clearly indicates that the soft coral natural product does not enhance excitability through cyclooxygenases, activation of a pertussis toxin G-protein or via protein kinase C.

### Actions of 3-carboxy-1-methyl pyridinium on cultured DRG neurones

**Figure 13:**
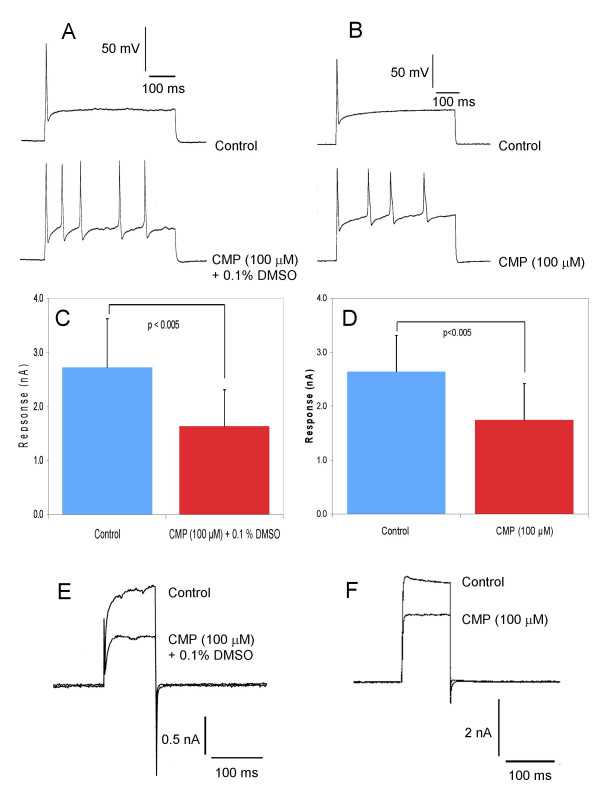
The presence or absence of DMSO has no effect on the actions of synthetic 3-carboxy-1-methyl pyridinium (CMP) on DRG neurones. A) Records of action potentials showing single action potential firing under control conditions and multiple firing induced by CMP (100 μM) in the presence of 0.1% DMSO. B) Records of action potentials showing single action potential firing under control conditions and multiple firing induced by CMP (100 μM) in the absence of DMSO. At rest both neurones were held at -70 mV C) Bar chart showing the significant decrease in the mean voltage-activated K^+ ^current (holding potential -70 mV, clamp potential for current activation +60 mV) evoked by CMP (100 μM) in the presence of 0.1% DMSO. D) Bar chart showing the significant decrease in the mean voltage-activated K^+ ^current (holding potential -70 mV, clamp potential for current activation +60 mV) evoked by CMP (100 μM) in the absence of DMSO. E & F) Current traces under voltage clamp showing the decrease in K^+ ^current produced by CMP.

The identification of 3-carboxyl-1-methyl pyridinium (CPM) as an active component in samples from *Sarcophyton glaucum *and *Lobophyton crassum *but not *Sinularia leptoclados *resulted in testing a synthetic sample of this compound. In our initial studies, we applied CPM in the presence of 0.1% DMSO to keep the recording conditions the same as with the soft coral extracts. Tests were also conducted in the absence of DMSO and it was found that the presence or absence of DMSO had no bearing on the results. Although 100 μM CPM attenuated spike frequency adaptation and induced multiple firing (Fig. 13A &13B), some features of the biological activity seen with the natural product samples were not seen with the synthetic compound. These included action potential prolongation and depolarizing drift in the electrotonic potential. This may be explained by the lower level of K^+ ^current inhibition seen with 100 μM CPM compared with 100 μg/mL crude sample. In the presence and absence of 0.1% DMSO, CMP inhibited the K^+ ^current at +60 mV by 51 ± 9 % (n = 6) and 42 ± 13 % (n = 5; Fig. 13C &13D). These data suggest that DMSO is not a factor in determining the actions of CPM. Raising the concentration of CPM to 1 mM resulted in greater inhibition of the K^+ ^current at +60 mV to 82 %. This compares with the level of inhibition of 90 % produced by 100 μg/mL of crude sample. From this we conclude that enhanced excitability of DRG neurones was caused by CPM, which is a major component in the crude samples from *Sarcophyton glaucum *and *Lobophyton crassum*. We estimate that the soft coral extract contained ~7% CPM, which would give approximately 50 μM CPM in the test dose of 100 μg/mL. Clearly there is an apparent anomaly between the time courses of the currents and amplitude of the responses observed when the actions of the soft coral samples and CPM from a synthetic source are compared. This discrepancy is not due to the presence of DMSO and would appear to result from an additional as yet unidentified natural product or products present in the samples. These other substances may enhance delivery and/or action of CPM.

## Conclusion

The crude samples from *Sarcophyton glaucum *and *Lobophyton crassum *but not *Sinularia leptoclados *gave NMR spectra that had common peaks in the region 7–9 ppm. The presence of a common natural product in two species of soft coral but not a third may result from biosynthesis in soft coral tissues but could also be the result of metabolism in symbiotic organisms common to *Sarcophyton glaucum *and *Lobophyton crassum*. However, all three species studied have symbiotic Zooxanthellae and the biosynthesis of CPM may reflect common ancestry. The optimum defense theory predicts that organisms with any sort of mechanical defense will not be vulnerable to predators and consequently lack chemical defenses. Thus, we suggest that *Sinularia *that is heavily encrusted and is highly packed with dense spicules does not produce CPM: while the fleshy flourishing *Lobophyton *and *Sarcophyton *samples produce defences that might include CPM for protection. Phylum Cnidaria, that include soft corals, may gain a level of defence from potential vertebrate predators by increasing excitability and producing abnormal firing patterns in sensory neurones. In certain Hydrozoa, relatively low concentrations of trigonelline (0.1–10 μM; 20 hours) antagonised larval metamorphosis induced by Cs^+ ^enriched seawater [30]. It is not clear if there is any relationship between the chemical control of development and chemical defence potentially provided (albeit at significantly higher concentrations) by the same compound.

Pyridinium compounds represent a biosynthetic starting point for pore forming alkylpyridinium chemical defences found in marine sponges [6-9,31]. They may also provide interesting evolutionary links or offer evidence for common symbiotic relationships between distinct organisms that have different chemical defences based around pyridinium salts.

The alkaloid CPM is not a novel compound and has a fairly wide distribution in the plant and animal kingdoms. It has been identified in a number of higher plant species [32] and in marine shellfish [33]. CPM acts as an osmoregulator in plants when they are exposed to excess salts, a function it may also have in some marine invertebrates. It also acts as a cell cycle regulator during the early growth of many legume root meristems [34]. In 1999, Tohda and colleagues [35] reported that CPM induced neurite outgrowth in human neuroblastoma cells, an activity that may be associated with electrophysiological changes and increased excitability.

Potassium channels are a highly diverse group of channels and are targets for a wide range of natural toxins from both marine and terrestrial organisms. Examples of toxins from marine invertebrates that inhibit voltage-activated K^+ ^conductances include sea anemone peptides (blood depressing substance BDS-I & II) [36,37], the coneshell toxin κ-conotoxin PVIIA [38] and latrunculin A from sponges and nudibranchs [39]. We have not assessed the selectivity of CPM for voltage-activated K^+ ^channel sub-types in DRG neurones for two reasons. Firstly, unlike many of the peptide toxins, CPM is not very potent. Secondly, DRG neurones are a heterogeneous population of neurones that express at least six different types of K^+ ^channels in distinct manners [40] and therefore would not provide a suitable assay preparation. Application of CPM increases electrophysiological excitability of DRG neurones. We predict from our study that it is a major component of the soft coral extracts that is responsible for changes in current kinetics and the inhibition of delayed rectifier K^+ ^channels. Additionally, CPM increases KCl-evoked Ca^2+ ^flux an action consistent with an increase in action potential firing. However, the intracellular Ca^2+ ^signals in part result from Ca^2+^-induced Ca^2+ ^release from intracellular stores and Ca^2+ ^release channels may also be modulated by CPM. Furthermore, altering the patterns of action potential firing in sensory neurones may provide mechanisms for chemical defence. The actions of the natural products and CPM was not restricted to a subpopulation of small diameter (C-fibre) neurones so a variety of sensory neurones may be effected.

## Methods

### Marine sample collection

Samples were collected from North of Sharm El-Sheikh, Egypt with the following coordinates (28° 07' 34" N and 34° 26' 28" E). Field observations were carried out by scuba diving. Underwater ecological observations of the reef profile where collections were recorded [41]. The percentage cover of the different substrates in the study area were estimated using line intercept transects and the percentage cover of each taxon was calculated. Substrate components were classified as hard corals, soft corals, dead corals (recognized by over growing algae), and others including plants (filamentous algae, calcareous algae, fleshly algae), associated fauna (molluscs, echinoderms and sponges), in addition to the 'dead substrate' component (sand and rock).

Directly after collection, the samples (60–70 g) were rinsed thoroughly in seawater and placed on ice for transfer to the laboratory and kept at -20°C until extraction. Each sample was split into two parts, one part (50 g) was used for the extraction and a second longitudinal section was used for the species identification.

Multiple extraction was done over three days using a mixture of 1:1 (v:v) methanol and dichloromethane. Following extraction at room temperature, under dark conditions the extracts were concentrated under reduced pressure to yield 459 mg of *Sarcophyton *extract.

Soft coral identification was accomplished by bleaching the tissue to remove debris and isolate the spicules. Morphological measurements and spicule examination were the key factors in identification [42].

Three species of soft corals were collected namely (*Sarcophyton glaucum, Sinularia leptoclados and Lobophyton crassum*). Voucher specimens are kept at the museum of the Marine Science Department at SuezCanal University and their index numbers are:

MSD OCT 10422 for *Sarcophyton *sample

MSD OCT 10442for *Sinularia *sample

MSD OCT 10452 for *Lobophyton *sample

### Chemical identification

UV spectra were measured on a Perkin-Elmer Lambda 15 UV/Vis spectrometer. ^1^H, ^13^C and all 2D NMR experiments were recorded on a Varian Unity INOVA 400 MHz spectrometer, in CD_3_OD (^1^H at 400 MHz and ^13^C at 100 MHz). A low-resolution electron ionisation mass spectrum was obtained using a Micromass Quattro II, and high-resolution mass data were obtained on a Finnigan MAT 95 XP. HPLC separations were carried out on a Phenomenex^® ^column (10 × 250 mm, RP-C18, 5 μm particle size) connected to an Agilent 1100 series binary pump and monitored using an Agilent photodiode array detector. Detection was carried out at 226, 264 and 320 nm. An authentic sample of 3-carboxy-1-methyl pyridinium (trigonelline hydrochloride) was purchased from Sigma Chemical Company (Dorset, UK).

Crude organic extracts of *Sarcophyton glaucum *and *Lobophyton crassum *were shipped to our laboratory at the University of Aberdeen for biological and chemical investigation and stored at -20°C until used. The close similarity of the ^1^H NMR spectra of both extracts especially in the region of δ_H _7–10 indicated the presence of at least one common constituent. Electrophysiological experiments showed that both extracts had the same effects on the electrophysiological actions on the cultured DRG sensory neurones from neonatal rats. This indicated that their common constituent(s) might be the responsible for such biological activity. NMR-, TLC- and biologically-guided fractionation of the crude extract of *Sarcophyton glaucum *was conducted to isolate such biologically active component(s). The crude extract was fractionated by reversed phase HPLC using a mixture of MeOH, water and TFA (80:20:0.05) as eluent. Fractions that showed the characteristic ^1^H NMR peaks of the common constituent in the region of δ_H _7–10 were pooled together, concentrated under reduced pressure and subjected to an HPLC using a linear gradient of MeOH (40–80 % in 20 min) in water at a flow rate of 2 mL/min. Further purification was carried out on the same HPLC column using two solvents: water (A) and methanol (B); starting with 0% MeOH and increasing to 10% B at 10 min and 15% B at 15 min with a flow rate of 2 mL/min. More purification was carried out using the same HPLC column with 100% water as eluent and a flow rate of 1 mL/min. Final purification was carried out using 0.1% TFA in water as eluent with a flow rate of 1 mL/min to afford 32 mg of 3-carboxy-1-methyl pyridinium.

The structure of 3-carboxy-1-methyl pyridinium was elucidated by a combination of NMR techniques, exact mass spectral determination, and comparison with the NMR data of related compounds in the literature.

### DRG neuron culture

Two-day old rats were decapitated and dorsal root ganglia were removed. DRG neurones were dissociated enzymatically (0.125% collagenase for 13 minutes and 0.25% trypsin for 6 minutes) and mechanically (trituration). Primary cultures of DRG neurones were plated on laminin-polyornithine coated coverslips and bathed in Ham's F-14 culture medium (Imperial Laboratories) containing horse serum (10%; Gibco), NGF (20 ng/mL; Sigma), NaHCO_3 _(14 mM), streptomycin (5000 μg/mL) and penicillin (5000 IU/mL). The cultures were maintained for up to two weeks at 37°C in humidified air with 5% CO_2_. Cultures were re-fed with fresh media after 5 days.

In one set of experiments, the DRG neurone cultures were pre-treated with pertussis toxin (500 ng/mL; for 18 hours) to ADP-ribosylate the α-subunits of certain G-proteins. This prevents pertussis toxin-sensitive G-protein being activated through a range of G-protein coupled receptors and potential effectors involved in increasing neuronal excitability [43].

### Patch clamp electrophysiology

The whole cell patch clamp recording method was used to investigate the actions of soft coral preparations on action potential firing and K^+ ^voltage-activated curents. DRG neurone cultures were studied using a patch pipette filling solution containing in mM: KCl, 140; EGTA, 5; CaCl_2_, 0.1; MgCl_2_, 2.0; HEPES, 10.0; ATP, 2.0. The pH and osmolarity of the patch pipette solutions were corrected to 7.2 and 310–320 mOsm.L^-1 ^with Tris and sucrose respectively. The NaCl-based extracellular solution containing in mM: NaCl, 130; KCl, 3.0; CaCl_2 _2.0; MgCl_2_, 0.6; NaHCO_3 _1.0, HEPES 10.0 glucose 5.0 and 0.1% DMSO. The pH and osmolarity of this extracellular bathing solution was corrected to 7.4 and 320 mOsmL^-1 ^with NaOH and sucrose respectively. The soft coral extracts were dissolved in DMSO (equivalent to 100 mg/mL). From these stock solutions, the test solutions were made up by dilution with extracellular solution so that they contained 0.1% DMSO. This concentration of DMSO vehicle will influence the electrophysiological properties of DRG neurones [44]. All experiments were conducted in the presence of 0.1% DMSO so that the actions of soft coral preparations could be assessed independently of the vehicle. Samples were applied to the extracellular environment by low-pressure ejection from a blunt pipette positioned about 50–100 μm away from the cell being recorded. This method allows a stable concentration of drug around a neurone to be achieved within ~10 s. For a series of experiments indomethacine (10 μM; 0.01% ethanol) or chelerythrine (3 μM) was applied extracellularly in the bathing solution and intracellularly via the patch pipette solution. After measurement of membrane potential, neurones were held at -70 mV with constant current injection and electrotonic potentials and action potentials were activated from this voltage.

All voltage-activated K^+ ^currents had scaled linear leakage and capacitance currents subtracted to obtain values for the net outward K^+ ^current. Data are given as mean ± standard error of the mean (SEM) values and statistical significance was determined using a paired or independent Student's *t *test as appropriate.

### Fura-2 Ca^2+ ^imaging

For Ca^2+ ^imaging, cultures were incubated for 1 hour in NaCl-based extracellular solution containing 0.01 mM fura-2AM (Sigma, 1 mM stock in dimethylformamide). The cells were then washed for 10–20 minutes with NaCl-based extracellular solution to remove the extracellular fura-2AM, this period allowed cytoplasmic de-esterification of the Ca^2+ ^sensitive fluorescent dye. The cells were constantly perfused with NaCl-based extracellular solution (1–2 mL/min) and viewed under an inverted Olympus BX50WI microscope. The fluorescence ratiometric images were taken with a KAI-1001 S/N 5B7890-4201 Olympus camera and the data obtained at excitation wavelengths of 340 nm and 380 nm. Images were viewed and analysed using OraCal pro, Merlin morphometry temporal mode (Life Sciences resources, version 1.20). The DRG neurones were stimulated with NaCl-based extracellular solution containing high K^+ ^(30 mM), which produced depolarization, activation of voltage-gated Ca^2+ ^channels and large transient increases in intracellular Ca^2+^. Three consistent transient increases in intracellular Ca^2+ ^could be obtained in a single experiment on cultured DRG neurones [45] and no more than 8.5 % variability was seen in any three-control responses from a single neurone [24]. The actions of soft coral samples (100 μg/mL) were investigated on the response to the second stimulus in DRG neurones and their actions on Ca^2+ ^transient amplitude, duration at 1/2 peak amplitude (W_50_) and total Ca^2+ ^flux were measured. The W_50 _value in seconds was determined by measuring the durations of the KCl-evoked Ca^2+ ^transients at the point of half their maximum amplitudes. This gives a standardized measurement of response duration. Total Ca^2+ ^flux is the change in fluorescence ratio × duration of response, the values are given with respect to Ca^2+ ^flux of 0.2 ratio units × 100 s. By cutting out and weighing records we obtained values for the areas under the curves for Ca^2+ ^transients. All experiments were conducted at room temperature and data are expressed as means ± SEM.

## Abbreviations

c.Lobo, crude sample from *Lobophyton crassum*

CPM, 3-carboxyl-1-methyl pyridinium (hydrochloride salt synthetic)

c.Sg, crude sample from *Sarcophyton glaucum*

c.Sl, crude sample from *Sinularia leptoclados*

DRG, Dorsal root ganglion.

NGF, Nerve growth factor

PKC, Protein kinase C

p.Sg, purified sample from *Sarcophyton glaucum*

## Authors' contributions

TT collected the soft coral specimens and participated in the biological characterization. WH and MJ carried out chemical analysis, purification and chemical identification of 3-carboxyl-1-methyl pyridinium. The electrophysiology and calcium imaging was conducted by WH, DW, KW, SN and RS. TT and RS conceived of the study and all authors read and approved the final manuscript.
